# The pre-hospital care conference

**DOI:** 10.1186/1757-7241-20-S1-I3

**Published:** 2012-03-22

**Authors:** Julian Thompson, Kate Crewdson

**Affiliations:** 1 Oxford, UK; 2 Bristol, UK

## 

The Pre-hospital Care Conference was the third and final day of the London Trauma Conference. The first speaker, Professor Malcolm Woollard, Director, Pre-hospital, Emergency & Cardiovascular Care Applied Research Unit, Coventry University, spoke on *Needle Decompression of the Chest – fact or fiction?* Professor Woollard’s comprehensive review of the epidemiology, appropriateness, effectiveness and risks of the procedure illustrated some interesting and important points. He argued that needle decompression of the chest is an infrequently performed procedure with currently taught techniques risking inappropriate use, failure and iatrogenic injury. Critically, multiple studies demonstrate that a standard 14G cannula of 4.5cm length will not penetrate the chest wall in many patients. Moreover, retrospective analysis of interventions undertaken reveals that the insertion site is often incorrect and frequently in the cardiac zone. He concluded that the intervention does have a role in the emergent management of trauma patients but emphasised that appropriate equipment should be used by practitioners with regular re-training in indications, technique and positioning.

Surgeon Commander Jason Smith, Royal Navy Consultant in Emergency Medicine, Derriford Hospital recently led a UK consensus group on treatment of crush injury and he spoke on *Crush Injury – is it More Than One Syndrome?* He answered this question by broadening the definition of the syndrome to ‘the systemic manifestation of muscle cell damage resulting from pressure or crush’ rather than the conventional perception of an injury caused by prolonged entrapment. He encouraged delegates to consider the diagnosis in a broad range of presentations and explained that the severity of the syndrome depends upon the magnitude and duration of the force and the bulk of muscle affected. This focus on damage to the sarcolemma explains how the same condition may develop from an instantaneous massive energy transfer or the prolonged application of a minor force to a large muscle bulk, such as that seen during prolonged immobility on a hard surface. The management of this condition adds an extra dimension to conventional trauma management and hyperkalaemia should be considered as a reversible cause of traumatic cardiac arrest when crush injury is suspected. Prevention of renal failure is paramount in the medical management of crush injury and, again counter to the current management of trauma patients, Surgeon Commander Smith advocated that aggressive crystalloid resuscitation be commenced in the pre-hospital setting. Reports from the management of crush injury in earthquake victims associate delay to fluid resuscitation as a risk factor for the development of renal failure. Mannitol and urine alkalisation should be considered although the evidence is limited, dated and largely anecdotal. Surgeon Commander Smith’s enthusiastic talk was interspersed with reverential pauses to admire spectacular photos of Royal Navy ships and helicopters – his prehospital workplace is rather more impressive than that of most of the conference delegates.

The controversial topic of *Compression only CPR* was addressed by Professor Kjetil Sunde, Oslo University Hospital, Norway in a fascinating and thoughtful analysis of some conflicting literature. There was a conventional central theme to this wide-ranging lecture: survival of cardiac arrest depends upon the chain of survival, consisting of early recognition, early CPR, early defibrillation and post resuscitation care. Professor Sunde’s unapologetic recycling of this familiar message was reinforced by the evidence that there is a 10 fold variation in reported OOHCA outcomes and that much can be done to improve the vital chain of survival. Evidence was presented to confirm that both early initiation and quality of CPR are critical and that the evidence demonstrating the superiority of compression only CPR over conventional 30:2 may reflect this. Non-CPR trained bystanders, particularly when guided by telephone instructions, are more likely to commence resuscitation and achieve adequate cardiac output with compression only CPR than when interrupted with reluctant attempts at ineffective ventilation. Despite this data, animal and human studies demonstrate that survival and neurological outcome are improved by effective ventilation and cerebral oxygenation. As a conclusion to the title of his talk, Professor Sunde advocated that CPR-trained bystanders and medical responders should continue conventional 30:2 CPR to optimise outcome. However his principle message was that the local variation in the outcome of OOHCA is unacceptable and that multiple studies demonstrate a simple and achievable solution to the problem: education of the general public in Basic Life Support. His thought provoking final comments were that randomised controlled trials on the minutiae of in-hospital care are irrelevant when compared to the potential for 10-fold increases in survival with education programmes.

The conference paused for coffee before a heretical session on pre-hospital interventions that challenged current recommendations by several international bodies. Professor David Lockey returned to the stage to convincingly advocate the *Emergency Surgical Airway* over needle cricothyroidotomy. Published cricothyroidotomy rates in emergency intubation vary from 0.3-1.0% [1,2], but current UK Difficult Airway Society guidelines are mildly ambiguous in the choice between needle and surgical technique in the ‘Can’t Intubate, Can’t Ventilate’ scenario, suggesting the use of a needle cricothyroidotomy before resorting to a surgical airway [3]. Professor Lockey argued that, when the airway is lost in the emergency prehospital situation, indecision and delay would increase the risk of hypoxic injury. He described a simple surgical technique with basic equipment that has been demonstrated to have a success rate of 100% when performed by prehospital physicians. His message was reinforced by evidence that needle cricothyroidotomy in the emergency scenario has a high failure rate and high rate of conversion to surgical cricothyroidotomy. A thorough review of the evidence was supported by the inclusion of the recent National Audit Project 4 from the Royal College of Anaesthetists, and the current European Resuscitation Council guidelines, both of which now advocate the use of surgical cricothyroidotomy over needle or cannula cricothyroidotomy [4].

Dr Dan Ellis, Consultant in Emergency, Critical Care and Pre-Hospital & Retrieval Medicine, Royal Adelaide Hospital, Australia talked passionately about p*re-hospital resuscitative thoracotomy* and immediately made a clear distinction between this procedure and in-hospital surgical thoracotomy. Pre-hospital resuscitative thoracotomy is performed only when a trauma patient is in cardiac arrest or an agonal state. Although historically manyl international authorities viewed the resuscitation of such patients as futile, London Air Ambulance’s data on pre-hospital resuscitative thoracotomy tells a very different story. Published international survival rates from traumatic cardiac arrest of all causes vary from less than 3 to 7.5%, [5], however pre-hospital resuscitative thoracotomy when performed according to local protocols in the context of penetrating injury has a survival rate of 18%. Australian, Japanese and US data of resuscitative thoracotomy in the ED also refute accusations of futility. Dr Ellis concluded that the evidence strongly supports the use of pre-hospital resuscitative thoracotomy in penetrating trauma with a likely duration of cardiac arrest of less than 15 minutes and advocated the clamshell technique. To add fuel to the fire of controversy he suggested extending the indication for the procedure to witnessed cardiac arrests from blunt trauma to achieve aortic control.

The Keynote Address of the pre-hospital care conference was given by Dr Arnd Timmerman, who has recently published the German society of anesthesiology and intensive care medicine airway management algorithm. Dr Timmerman spoke on the subject of p*re-hospital airway management* and his analysis of the evidence revealed that superficially reassuring intubation success rates hide a mass of morbidity and mortality behind reporting bias, selection bias, missing data and inadequate data collection. He suggested that the pre-hospital environment increases the difficulty of intubation and equipment limitation, particularly end tidal CO_2_ monitoring, result in significant rates of unrecognised oesophageal intubation with a high consequent mortality. After analysing the use of extraglottic (supraglottic) airway devices Dr Timmerman presented his recently published algorithm for pre-hospital airway management. He emphasised several key points: the need for strict indications for invasive airway management, mandatory use of end tidal CO_2_, education of pre-hospital clinicians and the consideration of extraglottic airways as a primary approach when difficult intubation is anticipated. This talk concluded a very challenging and stimulating morning and the delegates enjoyed lunch in the London summer sun.

The first afternoon session addressed large scale incidents and the delegates heard two fascinating and very different talks. Dr Malcolm Russell, Clinical Lead of Surrey and Sussex Air Ambulance and a member of the UK Search and Rescue team gave an inspiring lecture on *Medical Support for Natural Disasters – experience from New Zealand and Japan*. The logistics behind deploying a self-sufficient 65 man search and rescue team at short notice to anywhere in the world on a minuscule budget was fascinating. His description and photos of both events was humbling and distressing. Following the earthquake in Christchurch, New Zealand, the team was assigned to a collapsed office block where they worked day and night for over seven days to assess for signs of entrapped survivors. Sadly no one was recovered alive from the building, but the team made an important contribution by extricating 13 bodies and returning them to their families for burial. The disaster caused by the Japanese tsunami was on an entirely different scale and Dr Russell’s post-apocalyptic photos of obliterated towns were horrifying. It was startling how little medical care was required on both missions but that at these particular disasters the doctor’s role was one of maintaining the health of the team and adding an extra pair of hands to the rescue efforts.

Professor Pierre Carli started his talk entitled *Dealing with urban terrorism: the French approach* with an irreverent joke at his hosts’ expense; with Gallic confidence he proposed that the French were better than the British at cooking, perfume and seduction, and that he would demonstrate that they were also better at planning for the medical response to terrorism. At the end of his direct and practical lecture, the British in the audience were tempted to agree. Professor Carli explained that physicians lead routine French pre-hospital care both on scene and at the control centre, where a physician with several operators will coordinate medical resources. This physician-led command and control structure combined with domestic experience of bombings, shooting and toxic chemical release in France has resulted in clear and well rehearsed mass casualty plans. Pre-hospital doctors will run an advanced medical post on scene, including facilities for decontamination and advanced medical care, liaise closely with the in-hospital response and may trigger pre-agreed plans to triage stable patients to distant hospitals to allow efficient casualty flow. This system aims to avoid the uncontrolled overwhelming of local hospitals and the need for resource-intensive secondary transfer seen in incidents in Israel and USA where pre-hospital command and control is limited. This impressive coordination of major incident response is lead by regional ‘Prefects’, with considerable powers, who can monitor the flow of electronically tagged patients and available resources on a central computer.

After a short break, the final session of the conference reflected a couple of current social and medical vogues: the obesity epidemic and therapeutic hypothermia. Dr Miles Dalby, Consultant Cardiologist, Royal Brompton and Harefield Hospitals, London enthused about *Cath labs and cardiac arrests* with infectious passion for providing prompt primary angioplasty. Even the commonly encountered barriers to its provision such as normal post-arrest ECG or ongoing CPR were discounted. Realtime video of door to coronary stenting times demonstrated that the low technology solutions of multidisciplinary team-work and proactive leadership can deliver fantastic results. Dr Dalby also presented fascinating data demonstrating that mild therapeutic hypothermia induced around the time of primary angioplasty may reduce myocardial infarct size and improve contractility, providing yet another clinical application for this simplest of interventions.

Dr Matt Thomas, Consultant in Intensive Care at the University Hospitals, Bristol gave a very entertaining and practical lecture entitled *Big Problems in Pre-hospital Care.* The serious subtext to his talk was that the burgeoning population of obese patients present challenges at every stage of their pre-hospital and in-hospital care that require specific provision in terms of equipment and training. Practical tips for the safe management of the bariatric patient included the ramped position for intubation, early resort to supraglottic airway devices, the use of blood pressure cuffs on the forearm, and the tolerance of high airway pressures during ventilation. Dr Thomas presented surprising data to demonstrate that contrary to the expectation of many clinicians, obese patients have higher than the survival rates of non-obese patients with ischaemic heart disease, renal failure and on critical care. He argued that this ‘obesity paradox’ should dispel the medical prejudice that obese patients sometimes encounter.

A fascinating day concluded with a second talk from Professor Kjetil Sunde addressing *Pre-hospital Cooling for Medical Cardiac Arrest*. As with his earlier talk, Professor Sunde gave a measured analysis of the available evidence on timing, technique and duration for the initiation of therapeutic hypothermia post cardiac arrest. Whilst data strongly supports therapeutic hypothermia in comatose survivors of cardiac arrest, it is currently unclear whether pre-hospital or intra-arrest cooling will improve outcome further. Human studies have not satisfactorily addressed this question but a beautifully designed rat model of cardiac arrest demonstrated similar survival and neurological benefit when cooling was initiated between 0 and 4 hours post arrest and maintained for 24-48 [6]. Professor Sunde summarised by endorsing therapeutic hypothermia post cardiac arrest but could not demonstrate superiority for its initiation in the pre-hospital environment.

The London Pre-hospital Care Conference provided an entertaining and informative forum for the expertise and expanding evidence base in the field. Several speakers called for more International collaboration in research and major incident planning and, with its increasing numbers of delegates from many countries, the conference creates an opportunity for the spread of clinical excellence and for the genesis of such collaboration. Pre-hospital care remains a dynamic field of medicine and it was a privilege to hear some of the world’s leading exponents enthuse to an audience who may develop their own clinical practice in the light of new evidence.
